# Developing oncolytic *Herpes simplex virus type 1* through UL39 knockout by CRISPR-Cas9

**DOI:** 10.22038/ijbms.2020.43864.10286

**Published:** 2020-07

**Authors:** Saeedeh Ebrahimi, Manoochehr Makvandi, Samaneh Abbasi, Kayhan Azadmanesh, Ali Teimoori

**Affiliations:** 1 Infectious and Tropical Diseases Research Center, Health Research Institute, Ahvaz Jundishapur University of Medical Sciences, Ahvaz, Iran; 2 Abadan Faculty of Medical Science, Abadan, Iran; 3 Department of Virology, Pasteur Institute of Iran, Tehran, Iran; 4 Department of Virology, Faculty of Medicine, Hamadan University of Medical Sciences, Hamadan, Iran

**Keywords:** CRISPR-Cas9, Herpes simplex virus type 1, Oncolytic virus, Ribonucleotide reductase, UL39

## Abstract

**Objective(s):**

Oncolytic *Herpes simplex virus type 1* (HSV-1) has emerged as a promising strategy for cancer therapy. However, development of novel oncolytic mutants has remained a major challenge owing to low efficiency of conventional genome editing methods. Recently, CRISPR-Cas9 has revolutionized genome editing.

**Materials and Methods:**

In this study, we aimed to evaluate the capability of CRISPR-Cas9 to manipulate the UL39 gene to create oncolytic HSV-1. Herein, three sgRNAs were designed against the UL39 gene and transfected into HEK-293 cell line followed by infection with HSV-1 KOS.

**Results:**

After three rounds of plaque purification, several HSV-1 mutants were identified by PCR analysis and sequencing. One of these mutations in which 55 nucleotides were deleted resulted in a frameshift mutation that in turn produced a truncated protein with only 167 amino acids from 1137 amino acids. Functional analysis in Vero and primary fibroblast cells revealed that viral replication was significantly lower and plaque size was smaller in the HSV-1 mutant compared with HSV-1 KOS. Moreover, the relative amount of viral genome present in the supernatants of infected cells (Vero and primary fibroblast cells) with HSV-1 mutant was significantly decreased compared with those of HSV-1 KOS.

**Conclusion:**

Our data revealed that targeting UL39 with CRISPR-Cas9 could develop oncolytic HSV-1.

## Introduction

Oncolytic viral therapy has emerged as a novel and effective therapeutic strategy for cancer and promisingly entered human clinical trials against several cancers. Oncolytic viruses could be native or genetically manipulated viruses that could selectively replicate in tumor cells but not in normal cells. Genetically modified *Herpes simplex virus type 1* (HSV-1), the most commonly studied oncolytic virus, has been exploited as an effective vector in cancer therapy (1, 2). HSV-1, a member of the *Herpesviridae* is a common infectious agent in children and adolescents. HSV-1 has a relatively large (approximately 150 kb) dsDNA genome that encodes approximately 80 genes, half of which are not essential for viral replication. HSV-1 primary infection occurs in the epithelial cells of the mouth, lips, and eyes in the form of lytic phase. HSV-1 could also enter into the nerve terminals and in some cases could remain in a latent form (without replication and viral protein production) in the neurological system and could be reactivated (3). HSV-1, a neurotropic DNA virus has been regarded as an attractive candidate for oncolytic viruses due to their favorable features such as efficient replication rate, wide cell host range, as well as relatively large genome size. They are also relatively safe because their genome is not incorporated into the host genome. Moreover, the relatively large genome size of HSV-1 provides more targets to be manipulated to develop an appealing vector. Instantly, several HSV-1 vectors have been developed through manipulation of various genes such as UL23, UL39, γ34.5, and ICP47, which have key roles in pathogenicity and immunogenicity. Interestingly, it has been suggested that manipulation of these genes limits viral replication to dividing cells (1, 2, 4-7). The conventional strategies such as homologous recombination that construct a recombinant oncolytic HSV-1 are relatively inefficient, laborious, and time-consuming. Thus, it is crucial to develop novel strategies to construct recombinant oncolytic viruses. Several techniques have been introduced to edit viral genomes. One of the recent methods is the type II prokaryotic clustered regularly interspaced short palindromic repeats-associated CAS (CRISPR-Cas) system, which was originally derived from defense mechanisms in bacteria. CRISPR-Cas9 system is revolutionizing our understanding of fundamental issues in molecular biology and could play a key role in genome engineering of viruses. CRISPR-Cas9 system is a complex of a single guide RNA (sgRNA) that recognizes foreign or target genome and a Cas9 endonuclease that cuts target sequence. Following cleavage of the target sequence, a DNA double strand break (DSB) is formed that is repaired through either error-prone non-homologous end joining (NHEJ) or homology directed repair (HDR) system. During the repair process, random indels could be introduced at the cleavage site resulting in the destruction of the open reading frame and consequently inactivation of the target gene (8-14). Several studies have successfully used the CRISPR-Cas9 system to manipulate HSV-1 genome through deletion of multiple genes such as UL21, UL7, UL23, ICP0, and US8 (12, 15-20). The UL39 gene of HSV-1 encodes the large subunit of ribonucleotide reductase (RR protein), a protein complex that participates in viral DNA replication via converting ribonucleotides to deoxyribonucleotides. Previous studies have suggested that replication of viruses with mutation in this gene is highly dependent on ribonucleotide reductase activity, which is active in tumor cells while not in normal cells with limited dividing activity (21, 22). In the current study, we aimed to evaluate the CRISPR-Cas9 system capability to construct oncolytic HSV-1 through induction of UL39 mutation. 

## Materials and Methods

***Cell culture and viruses***

In this study, two cell lines including human embryonic kidney (HEK) -293 cell line and African green monkey kidney cell line (Vero), which were obtained from Pasture Institute of Iran, and primary human foreskin fibroblast (HFF) cells, which were prepared in our lab, were used (23). The cells were cultured in Dulbecco’s modified Eagle’s medium (DMEM, Gibco, Grand Island, NY, USA) supplemented with 10% fetal bovine serum (FBS; Gibco), 100 U/ml penicillin, and 100 g/ml streptomycin at 37 ^°^C with a humidified atmosphere containing 5% CO_2_. HSV-1 was obtained from Tarbiat Modares University, Tehran, Iran.

***Construction of sgRNA/Cas9 vectors***

Three sgRNAs targeting UL39 (listed in Table 1) were designed using the CRISPR-Cas9 design tool developed by the Zhang lab (http://crispr.mit.edu) and synthesized as DNA oligonucleotides. The annealing of each top-strand oligonucleotide to its corresponding bottom-strand oligonucleotide was performed (in a thermocycler, using 5 μl of each oligo at the concentration of 10 µM and 40 μl of water at either 95, 80, 70, 60, 55, or 45 ^°^C for 30 sec) and the dsDNA fragment was sub-cloned into pCas-Guide-EF1a-GFP CRISPR Vector (ORIGENE plasmid GE100018) between BamH1 and BsmBI restriction sites. The insertion of sgRNAs was evaluated via sequencing. 

***Cell transfection, infection and generation of UL39 mutant***

In order to generate HSV-1 mutant, the HEK-293 cell line was seeded in a 24-well plate and transfected when the confluency reached 80%. The transfection was carried out using 1 μg per well of either pCas guides UL39-eGFP or mock-targeting Cas9/sgRNA, which was facilitated by Turbofect reagent according to the manufacturer’s instructions (Thermo Fisher; Waltham MA, USA). After incubation for 5 hr at 37 ^°^C, the media was removed and the cells were gently washed with PBS and infected with HSV-1 KOS (WT) at multiplicity of infection (MOI) of 1. Supernatant was collected 48 hr post-infection (p.i.).

***Isolation and purification of mutant viruses by plaque purification***

Plaque assay was performed for culture supernatants obtained from transfected cells with UL39 Cas9/sgRNAs or mock Cas9/sgRNA. Vero cells were seeded into 6-well plates and incubated with complete DMEM in a humidified CO_2_ incubator at 37 ^°^C overnight. When the confluency reached 90-100%, the cells were infected by serially diluted supernatants (a 10-fold dilution was used) that were expected to contain mutated viruses. After 1 hr incubation at 37 ^°^C, the media was carefully aspirated. Cells were overlaid with equal volume of 2X DMEM and 1.5% agarose (Seakem LE Agarose, Lonza AG, Switzerland) and incubated for 72 hr to allow the formation of plaques. Plaque purification was repeated 3 rounds to separate a mutant virus from any wild-type virus contamination. Then, 20 single plaques were picked up using sterile Pasteur pipettes and placed in 500 µl of DMEM .Viruses were released by three cycles of freeze and thaw.

***PCR analysis and sequencing for detection of mutant progeny***

The isolated plaques obtained from plaque-purification were screened for possible deletion in UL39 by PCR and Sanger sequencing. Briefly, viral DNA was extracted using a High pure viral nucleic acid kit (Qiagen, Hilden, Germany). The genomic region in the vicinity of the CRISPR-Cas9 target site was PCR-amplified through following primers to obtain a 652 bp fragment.

UL39-sense: GTTGCGGTGACAAACATCGG 

UL39-antisense: CGGTTCACGGCATCTCCCAGAA.

Then, the PCR products were loaded on a 2% agarose gel and were purified using the universal DNA purification kit (Favorgen, Taiwan). Finally, CRISPR-Cas9-mediated mutagenesis in the UL39 locus was determined by Sanger sequencing.

***In-vitro growth kinetics***

The isolated plaques, which contain the HSV-1 mutants or HSV-1 KOS (WT), were used to infect Vero and HFF cells with 90% confluency at MOI of 0.01. After 1 hr incubation which allows viruses to be absorbed, the viruses were removed, and infected cells were washed twice with sterile PBS. Then, Vero cells were overlaid with the maintenance medium (2% fetal bovine serum) and HFF cells were incubated with serum free medium at 37 ^°^C. After, 24, 48, and 72 hr the cells and their corresponding media were collected together, and subjected to three cycles of freeze-thaw. Consequently, virus titers were determined using plaque assay in Vero cells.

***Quantitative PCR (qPCR)***

Real Time quantitative Polymerase Chain Reaction (RT-qPCR) was performed to quantify the relative amount of HSV-1 genomic DNA in the supernatants from HSV-1 infected Vero and HFF cells. Viral genomic DNA was extracted as described above, and qPCR was performed on a lightcycler 96 (Life Technologies Roche Life Science, Basel, Switzerland) using RealQ plus Master Mix and specific Probe for HSV-1 gB gene (Ampliqon, Denmark). HSV-1 gB gene was amplified using:

Forward primer 5’-GGAACCTGGTCATCCTTTGC-3’, Reverse primer 5’-ACGTGCATGGACCGGTTAAT-3’, and FAM-BHQ1 TaqMan probe 5’-CGCAGGCAC TCGTACTGCTCGCT-3’.

## Results

***Generation of HSV-1 UL39-mutant using the CRISPR-Cas9 system***

**Figure 1:**
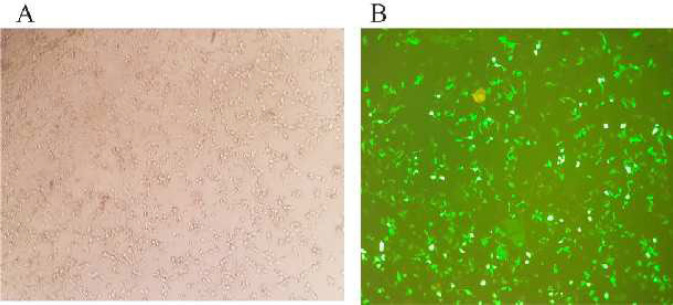
Transfection efficiency of plasmids in HEK-293 cells

Three sgRNAs (sgRNA-0, sgRNA-1, and sgRNA-2) were designed against three specific regions within the UL39 gene. These specific regions included 554–573 (0), 485–504 (1), and 407-426 (2). Initially, the sgRNAs were introduced into HEK-293 cells, 5 hr later, the transfected cells were infected with HSV-1 KOS (WT) at MOI=1. Cell supernatants were collected 48 hr p.i. The transfection efficiency was evaluated using an invert fluorescent microscope at 48 hr post-transfection (Figure 1). 

**Figure 2:**
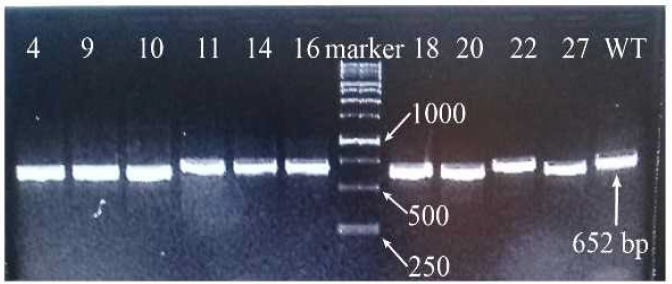
Genome PCR of isolated clones. Several clones (4, 9, 10, 11, 14, 16, 18, 20, 22, 27) were randomly isolated and target site of their UL39 gene was amplified via PCR and visualized via Electrophoresis. Each band represents a clone. The band of some clones locates lower than that of the wild type

Then, in order to isolate HSV-1 clones mediated by the CRISPR-Cas9 system, the culture supernatants containing HSV-1 mutant were purified using three rounds of plaque purification assay in Vero cells. Finally, 20 clones were randomly isolated and their genomic DNA was extracted. The HSV-1 genome around UL39 was amplified using specific primers and consequently sequenced to confirm CRISPR-Cas9 - derived mutations. As seen in Figure 2, PCR product of some clones were shorter in length compared to that in HSV-1 KOS (WT), suggesting that deletion might be introduced.

**Figure 3:**
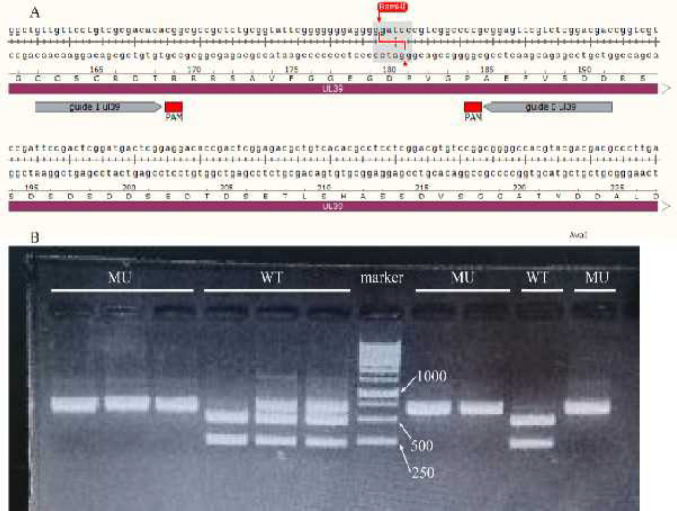
Identification of CRISPR-Cas9 derived-indel mutations by restriction enzyme assay

BamH1 enzymatic site was located between cleavage sites of gRNA-1, 0. It was expected that CRISPR-Cas9 destroys the BamH1 enzymatic site; therefore, we evaluated the restriction pattern of PCR product (650bp) of these clones through restriction digests by BamH1 enzyme (Figure 3). 

**Figure 4:**
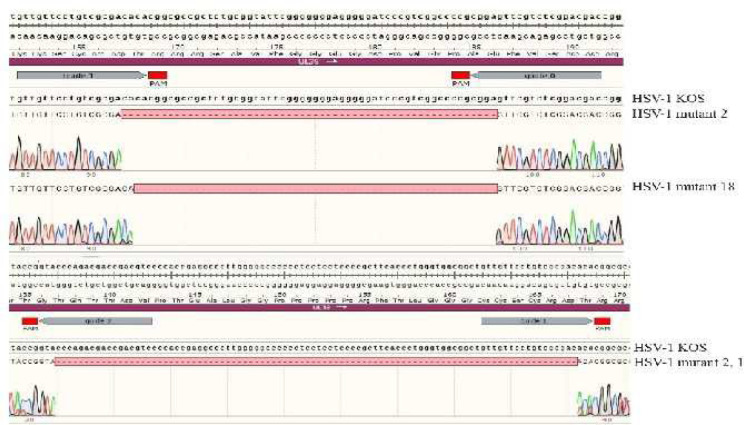
Sequencing analysis of isolated clones

The sequencing data indicated that all three sgRNAs could lead to site specific cleavage in the UL39 gene. Statistically, mutations were observed in 9 of the 20 clones (45 %); while, the UL39 gene remained intact in 11 clones (55%). Further characterization of the mutant clones via sequencing analysis revealed that sgRNA 0 and 1 could lead to 2 different mutations. In one of the mutations that was observed in the majority of mutant clones (clones 18, 20, 4, 9, 27, and 10) 55 nucleotides were deleted; while, in another mutation which was detected in one mutant clone (Clone 2) 57 nucleotides were missing. Moreover, sgRNA 1 and 2 induced a mutation only in one clone (Clone 2-1) in which 92 nucleotides were deleted (Figure 4).

**Figure 5:**
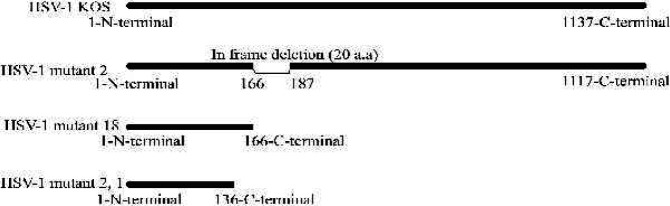
Schematic representation of RR protein in HSV-1 KOS (WT), HSV-1 mutant 2, HSV-1 mutant 18 and HSV-1 mutant 2- 1

These mutations might lead to various lesions such as frame shift mutations and premature stop codons; these lesions in turn could result in truncated forms of UL39. Further analysis of these mutations showed that 55 or 92 deleted nucleotides could lead to frame shift in the UL39 gene. Moreover, bioinformatics analysis showed that these mutations might generate only the initial 167 (corresponding to the 55 deleted nucleotides) and 123 (corresponding to the 92 deleted nucleotides) amino acids of the RR protein. On the other hand, the 57 missing nucleotides from the UL39 gene, resulted in the removal of 20 initial amino acids in the protein reading frame (Figure 5). Finally Two mutant viruses (HSV-1 mutant 18, HSV-1 mutant 2) produced by sgRNAs 0, 1 were chosen for further analysis.

***Replication kinetics of HSV-1 UL39 mutant***

**Figure 6:**
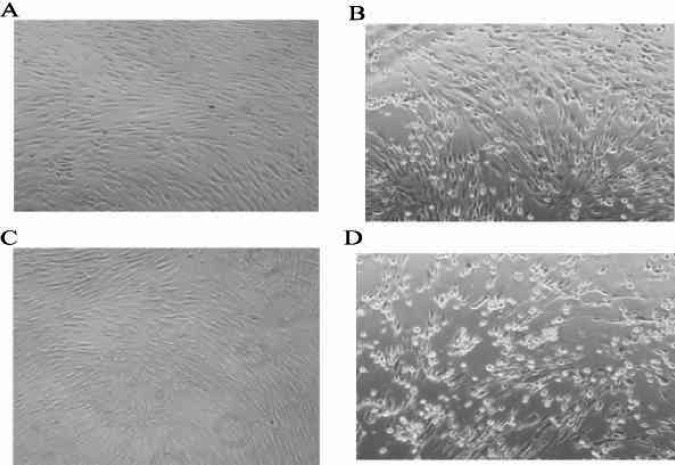
HFF cell cultures infected with the HSV-1 mutants 18, 2 and HSV-1 KOS (WT). Control non infected cell cultures (A) and cytopathic effect HSV-1 KOS (WT) at 72h (B), cytopathic effect HSV-1 mutants18 at 72h (C) and cytopathic effect HSV-1 mutants 2 at 72 hr (D) post-infection. Unstained fresh cultures (40X))

**Figure 7:**
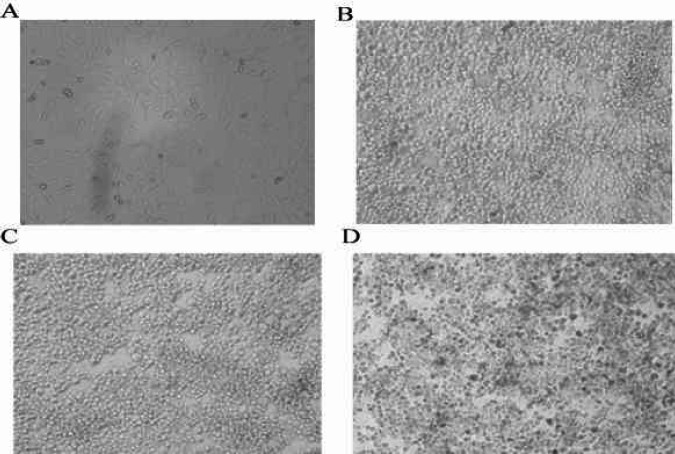
Vero cell cultures infected with the HSV-1 mutants 18, 2 and HSV-1 KOS (WT). Control non infected cell cultures (A) and cytopathic effect HSV-1 KOS (WT) at 72 hr (B), cytopathic effect HSV-1 mutants 2 at 72 hr (C) and cytopathic effect HSV-1 mutants18 at 72h (D) post-infection. Unstained fresh cultures (40X))

To identify the replication potential of UL39 mutant viruses compared with HSV-1 KOS (WT), the growth characteristics were determined in Vero and HFF cells. Cultured HFF and Vero cells were infected with HSV-1 KOS (WT), HSV-1 mutant 18, and HSV-1 mutant 2 at MOI of 0.01. Progeny viruses were harvested 24, 48, and 72 hr p.i. (Figures 6 and 7) and tittered with the standard plaque assay on Vero cells to determine growth properties.

**Figure 8:**
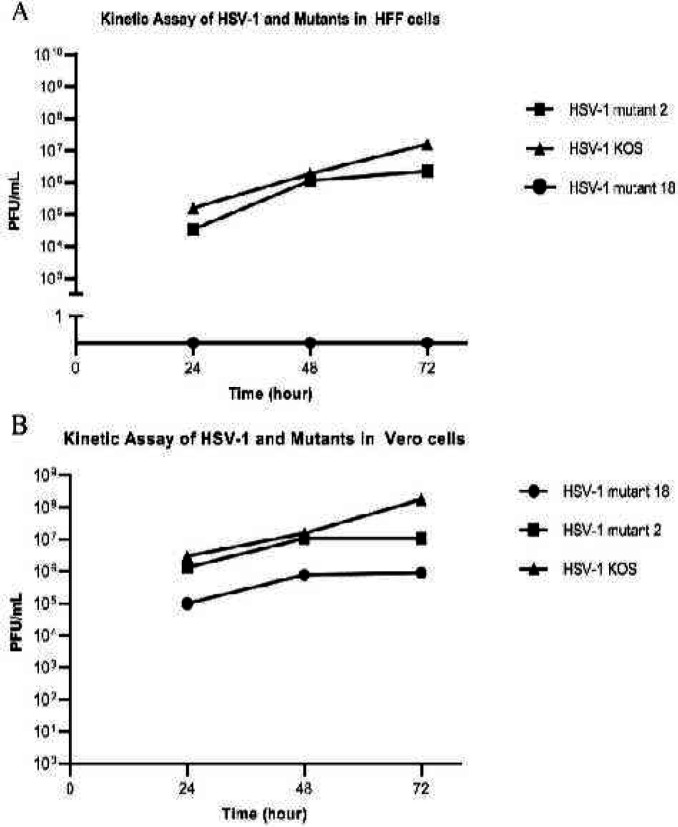
Viral growth kinetics in HFF (A) and Vero (B) cells. Growth kinetics of HSV-1 KOS (WT), HSV-1 mutant 18 and HSV-1 mutant 2 in HFF and Vero cells were assessed. When confluency reached 90%, the cells were infected with viruses at a multiplicity of infection of 0.01. Supernatants were collected at 24, 48, and 72 hr post infection and consequently the viral titers were determined by plaque assays on Vero cells

As seen in Figure 8, in HFF cells HSV-1 KOS (WT) grew at a higher rate than HSV-1 mutant 2 and HSV-1 mutant 18. HSV-1 mutant 2 titer was 0.5 Log unit lower than that of HSV-1 KOS (WT) at 24 hr p.i., 1 log unit lower than that of HSV-1 KOS (WT) at 72 hr p.i., and the same as HSV-1 KOS (WT) titer at 48 hr p.i. HSV-1 mutant 18 did not grow as well at any time in HFF cells. On the other hand, in Vero cells, the titer of HSV-1 KOS (WT) and HSV-1 mutant 2 was approximately equal at 24 and 48 hr p.i.; while, HSV-1 mutant 2 reached approximately 1 log unit lower than that of HSV-1 KOS (WT) titer at 72 hr p.i. Additionally, HSV-1 mutant 18 titer was about 1.5 logs unit lower than those of HSV-1 KOS (WT) at 24 and 48 hr p.i., and 2 logs unit lower than that of HSV-1 KOS (WT) at 72 hr p.i. The results demonstrated that the growth rate of HSV-1 mutant 18 was significantly lower than those of HSV-1 KOS (WT) and HSV-1 mutant 2 in both cells.

**Figure 9:**
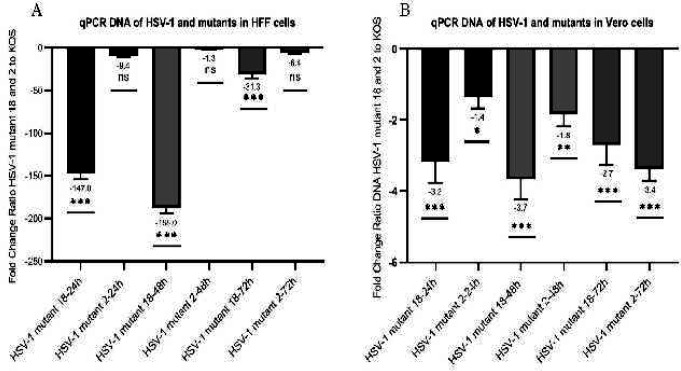
Relative amount of viral DNA in HFF (A) and Vero (B) cells

The replication potential of the HSV-1 mutant viruses compared with HSV-1 KOS (WT) was further evaluated using qPCR. To this end, the viral genomes present in the supernatants of infected cells (Vero or HFF) with either HSV-1 mutant 2, HSV-1 mutant 18, or HSV-1 KOS (WT) were quantified via qPCR. As presented in Figure 9, the amount of viral DNA present in the supernatants of infected HFF cells was decreased for HSV-1 mutant 18 (147-fold at 24 hr p.i., 188-fold at 48 hr p.i., and 31-fold at 72 hr p.i.) relative to the level for HSV-1 KOS (WT). Likewise, the relative amount of viral DNA present in the supernatants of infected HFF cells with HSV-1 mutant 2 was declined (9.4-fold at 24 hr p.i., 1.3-fold at 48 hr p.i., and 6.4-fold at 72 hr p.i.). As with infected HFF cells, the amount of viral DNA from the supernatant of infected Vero cells with HSV-1 mutant 18 was declined (3.2-fold at 24 hr p.i., 3.7-fold at 48 hr p.i., and 2.7-fold at 72 hr p.i.) compared to the level for HSV-1 KOS (WT). Moreover, the relative amount of viral DNA present in the supernatants of infected Vero cells with HSV-1 mutant 2 was decreased (1.4-fold at 24 hr p.i., 1.8-fold at 48 hr p.i., and 3.4-fold at 72 hr p.i.).As expected, we observed a significant drop in the amount of viral particles present in the supernatants of infected cells with HSV-1 mutant 18 compared with those of HSV-1 KOS (WT) and HSV-1 mutant 2. 

***Characterization of plaque size***

**Figure 10:**
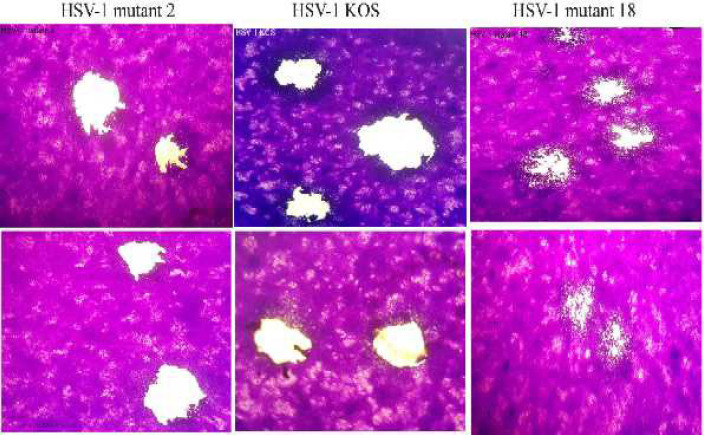
Plaque features of HSV-1 mutants18, 2 and HSV-1 KOS (WT). Plaques of HSV-1 mutant 18 were significantly smaller than those of HSV-1 mutant 2 and HSV-1 KOS (WT). Vero cells were infected with HSV-1 KOS (WT) and HSV-1 mutants 18, 2 then overlaid with 1.5% agarose. 3 days post infection, plaques were fixed with formaldehyde10 % and stained with 1% crystal violet solution

As shown in Figure 10, there were distinct differences in the plaque morphologies between HSV-1 mutant 18 and HSV-1 KOS (WT) in Vero cells. The plaque size of HSV-1 mutant 18 was significantly smaller than that of HSV-1 KOS (WT) which further supported the lower growth rate of HSV-1 mutant 18. 

**Table 1:** Overview of the synthesized sgRNA sequences for oligonucleotides used to produce UL39 sgRNAs

**sgRNA Name**	**Top strand (5'-3')**	**Bottom strand (5'-3')**
**0**	gat cgG TCG TCC GAG ACG AAC TCC Gg	aaa acC GGA GTT CGT CTC GGA CGA Cc
**1**	gat cgG TTG TTC CTG TCG CGA CAC Ag	aaa acT GTG TCG CGA CAG GAA CAA Cc
**2**	gat cgG ACG TCG GTC GTC TGG GTA Cg	aaa acG TAC CCA GAC GAC CGA CGT Cc

Small letters indicate restriction enzyme sites for BamHI and BsmBI; capital letters indicate the complement sequences of UL39 in HSV-1

A: Light microscopy analysis of HEK-293 cells transfected with 3 pCas-Guide-EF1a-GFP CRISPR plasmids. B: The transfected cells with the plasmids were imaged through fluorescence microscopy. The cells were imaged at 48 hr post-transfection

A: Schematic diagram shows that the BamH1 restriction site is located between the sgRNA-1 and sgRNA-0 cleavage sites. SgRNA-1 and sgRNA-0 destroy the BamH1 restriction site. B: BamH1 enzyme produces two bands (470 and 225 nt) in wild clones (WT) and one band (640 nt) in mutant clones (MU)

Schematic representation of the target sequences on UL39 in HSV-1 KOS (WT) parallel with those of HSV-1 mutant 2, HSV-1 mutant 18, and HSV-1 mutant 2-1

Amino acid Position of the deleted sequence is shown. HSV-1 mutant 2 includes an in-frame mutation in which 20 amino acids are deleted on RR protein; while, HSV-1 mutant 18 and HSV-1 mutant 2-1 have truncated proteins with only 167 and 136 amino acids respectively from 1137

A: Viral DNA was significantly decreased in HSV-1mutant 18 compared to that of HSV-1 KOS (WT). While, there is no significant difference between HSV-1mutant 2 and HSV-1 KOS (WT) in HFF cells. B: Viral DNA was significantly decreased in HSV-1 mutant 18 compared to that of HSV-1 KOS (WT). While, there is no significant difference between HSV-1mutant 2 and HSV-1 KOS (WT) in Vero cells

Ns: Not significant, *P<* 0.0001 ***, *P<* 0.001 **, *P<* 0.05- 0.01 *

## Discussion

Oncolytic HSV-1 has emerged as a promising vector to target and eliminate different tumors. An appealing feature that makes HSV-1 a promising oncolytic virus is its genome, which encodes many genes whose manipulation might confer safety and/or tumor targeting specificity (1, 2, 6, 7). UL39 has been suggested as an appropriate target gene whose deletion might enhance its selectivity to tumor cells. The UL39 gene has a key role in viral replication because it converts ribonucleotides to deoxyribonucleotides thereby providing dNTP pools for DNA synthesis. Therefore, it has been suggested that HSV-1 mutants with the UL39 gene deleted could replicate only in dividing cells such as tumor cells that overexpress RR (4, 5, 21). 

Herein, we showed that the selected region on the HSV-1 genome could be successfully manipulated via three sgRNAs. Initially, we employed one sgRNA to target the UL39 gene on the HSV-1 genome; however, because of the lower efficiency obtained we used multiple sgRNAs to increase editing efficiency. In agreement with this observation, various studies indicated that the combination of multiple sgRNAs could increase the deletion probability of the target region via CRISPR-Cas9 system (9, 24-26). Generally, the CRISPR-Cas9-mediated DNA cleavage might be repaired via the error-prone non-homologous end joining (NHEJ) repair pathway, which probably inserts or deletes 5 to 9 nucleotides to the cleaved site. So far, the detection of such indels is mainly carried out via Sanger sequencing of PCR products of the target region. Indeed, identification of CRISPR-Cas9 effects with conventional techniques such as PCR is of great interest (27); therefore, in this study, the distance between each sgRNA with the next sgRNA was considered for 50 nucleotides. In the case of cleavage with a combination of multiple sgRNAs simultaneously, the digested region could be simply identified via PCR and observed on agarose gel. Therefore, the initial identification of novel mutant viruses from different viral clones could be easily and quickly detected using PCR. 

Following target site cleavage by Cas9/sgRNAs, the random deletion of nucleotides upstream of the PAM sequence resulted in the induction of three different mutations in the UL39 gene. One of these mutations was HSV-1 mutant 18 in which 55 nucleotides that located within the 502-557 region were deleted. This deletion might lead to induction of frame shift mutation in the UL39 open reading frame which in turn produced only 167 amino acids from 1137 amino acids of RR protein. Therefore, this mutation might consequently lead to truncated RR protein. Consistently, Chen *et al.* suggested that frameshift indels are mainly prone to generate loss-of-function mutations, which are translated into truncated non-functional proteins (28). The previous studies showed that deletion of the UL39 gene could decrease the titer of recombinant viruses (4, 5, 21, 29, 30). Analysis of the new viruses in Vero and HFF cells revealed that the replication efficacy of HSV-1 mutant 18 was declined and the plaque morphology was altered compared with the HSV-1 KOS (WT). Moreover, our data indicated that deletion of the UL39 gene significantly decreased viral loads produced by infected Vero cells. As indicated, the viral titer was 1.5-2 log unit lower for HSV-1 mutant 18 in comparison with HSV-1 KOS (WT). Besides regulating viral replication, RR protein could inhibit apoptosis thereby mutation of the UL39 gene could induce apoptosis (31, 32) and, probably, halt optimal viral propagation and this might be an explanation for the reduction of HSV-1 mutant 18 titer in Vero cells. Several studies showed that the RR protein was not crucial for virus replication in dividing cells, while, it was vital for viral growth in serum starved or neuronal cells with limited dividing capability (4, 5, 21, 29, 30). Consistently, our data indicated that in serum-starved HFF cells in which HFF cells were in the G0 phase of the cell cycle were infected with HSV-1 mutant 18, viral growth was inhibited, no plaques were observed and DNA synthesis was compromised. However, HSV-1 mutant 18 could replicate in HFF cells cultured in 15 % serum (data not shown). Therefore, this data demonstrated that HSV-1 with UL39 mutation could replicate in dividing primary cells or tumor cells with high levels of dNTPs. Besides, the smaller plaque size of HSV-1 mutant 18, further highlighted the notion that the viral growth rate was significantly lower in HSV-1 mutant 18 compared with HSV-1 KOS (WT).

Interestingly, we demonstrated that 2 concurrent sgRNAs might induce a mutation which is not functionally influential. Instantly, in HSV-1 mutant 2, 2 sgRNAs deleted 50 nucleotides without changing the reading frame of the UL39 gene. Interestingly, this deletion did not induce significant changes in the replication kinetics of HSV-1 mutant 2 and plaque phenotype compared to those of HSV-1 KOS (WT). Therefore, this method can be exploited to delete specific domains in proteins without interfering in canonical protein functions. 

## Conclusion

The CRISPR-Cas9 system has become one of the powerful and reliable methods to modify HSV-1 genome with the aim of generating recombinant viruses. It is more effective, robust, low cost and simple than other conventional methods such as homologous recombination method and bacterial artificial chromosome (BAC) plasmid system. According to the higher efficiency of CRISPR-Cas9 system, we were able to construct HSV-1 attenuated mutants, which could facilitate the development of oncolytic vectors.
